# Modified endoscopic submucosal dissection with enucleation for treatment of gastric subepithelial tumors originating from the muscularis propria layer

**DOI:** 10.1186/1471-230X-12-124

**Published:** 2012-09-14

**Authors:** Yin-Yi Chu, Jau-Min Lien, Ming-Hung Tsai, Cheng-Tang Chiu, Tse-Ching Chen, Kuo-Ching Yang, Soh-Ching Ng

**Affiliations:** 1 Department of Gastroenterology, Shin Kong Wu Ho-Su Memorial Hospital, Taipei, Taiwan; 2 Department of Gastroenterology and Hepatology, Chang Gung Memorial Hospital, Taoyuan, Taiwan. College of Medicine, Chang Gung University, Taoyuan, Taiwan; 3 Department of Internal Medicine, Chang Gung Memorial Hospital, Keelung, Taiwan. College of Medicine, Chang Gung University, Taoyuan, Taiwan; 4 Department of Pathology, Chang Gung Memorial Hospital, Taoyuan, Taiwan. College of Medicine, Chang Gung University, Taoyuan, Taiwan

**Keywords:** Endoscopic submucosal dissection, Gastrointestinal stromal tumors, Endoscopic ultrasonography

## Abstract

**Background:**

Gastric subepithelial tumors are usually asymptomatic and observed incidentally during endoscopic examination. Although most of these tumors are considered benign, some have a potential for malignant transformation, particularly those originating from the muscularis propria layer. For this type of tumor, surgical resection is the standard treatment of choice. With recent advent of endoscopic resection techniques and devices, endoscopic submucosal dissection (ESD) has been considered as an alternative way of treatment. The aim of this study is to demonstrate the feasibility of a modified ESD technique with enucleation for removal of gastric subepithelial tumors originating from the muscularis propria layer, and to evaluate its efficacy and safety.

**Methods:**

From November 2009 to May 2011, a total of 16 patients received a modified ESD with enucleation for their subepithelial tumors. All tumors were smaller than 5 cm and originated from the muscularis propria layer of the stomach, as shown by endoscopic ultrasonography (EUS). The procedure was conducted with an insulated-tip knife 2. Patient’s demographics, tumor size and pathological diagnosis, procedure time, procedure-related complication, and treatment outcome were reviewed.

**Results:**

Fifteen of the sixteen tumors were successful complete resection. The mean tumor size measured by EUS was 26.1 mm (range: 20–42 mm). The mean procedure time was 52 minutes (range: 30–120 minutes). Endoscopic features of the 4 tumors were pedunculated and 12 were sessile. Their immunohistochemical diagnosis was c-kit (+) stromal tumor in 14 patients and leiomyoma in 2 patients. There was no procedure-related perforation or overt bleeding. During a mean follow up duration of 14.8 months (range: 6–22 months), there was no tumor recurrence or metastasis.

**Conclusions:**

Using a modified ESD with enucleation for treatment of gastric subepithelial tumors originating from the muscularis propria layer and larger than 2 cm, complete resection can be successfully performed without serious complication. It is a safe and effective alternative to surgical therapy for these tumors of 2 to 5 cm in size.

## Background

Subepithelial tumors are an uncommon entity of upper gastrointestinal (GI) tract with an estimated overall prevalence of 0.3% [1]. Most of the gastric subepithelial tumors are asymptomatic and observed incidentally during endoscopic examination. Although these tumors are considered benign, some have a potential for malignant transformation, particularly those originating from the muscularis propria layer [2]. This latter group of subepithelial tumors can be further classified as gastrointestinal stromal tumors (GISTs), myogenic tumors, and neurogenic tumors. The most common one is GIST. For symptomatic larger (> 3 cm) GISTs, surgical resection is the current standard treatment of choice. It has been recommended that endoscopic surveillance every 6–12 months is sufficient for those smaller GISTs of less than 2 cm in size [3], although natural history of these smaller tumors has never been elucidated. Miettinen et al. [4] followed 1055 patients with gastric GISTs of between 2 to 5 cm in size after surgical removal and observed a 1.9-16% risk of metachronous metastasis or tumor-related death.

In recent years, a newly-developed technique, endoscopic submucosal dissection (ESD), has been successfully introduced for treatment of early gastrointestinal cancer [5]. This technique makes complete resection of large mucosal lesion feasible. Few endoscopists have attempted to use ESD technique for management of the subepithelial tumors originating from the muscularis propria layer. The aim of this study is to demonstrate the feasibility of a modified ESD technique with enucleation for removal of larger subepithelial tumors originating from the muscularis propria layer, and to evaluate its efficacy and safety.

## Methods

A retrospective chart review was conducted. A total of 16 patients (6 men and 10 women; mean age, 51.9 years old, range: 35–65) received the treatment between November 2009 and May 2011. All had endoscopy, EUS, and computed tomography (CT) of abdomen before ESD. Patients who had tumor of greater than 5 cm in size as measured by EUS or tumor associated with ulcer, who presented with overt tumor bleeding, or distant metastasis were excluded. EUS was performed with a mechanic radial-scanning echoendoscope (GF-UMQ 260, 7.5-12 MHz; Olympus Optical, Tokyo, Japan), an electronic radial-scanning echoendoscope (GF-UE 260, 5–10 MHz; Olympus Optical, Tokyo, Japan), or an ultrasonic miniature catheter probe (UM-2R, 12 MHz; Olympus Optical, Tokyo, Japan). All subepithelial tumors were diagnosed as arising from the muscularis propria layer of the stomach. Approval for this retrospective study was obtained from our institutional review board (CGMH 101-0432B).

**Figure 1:**
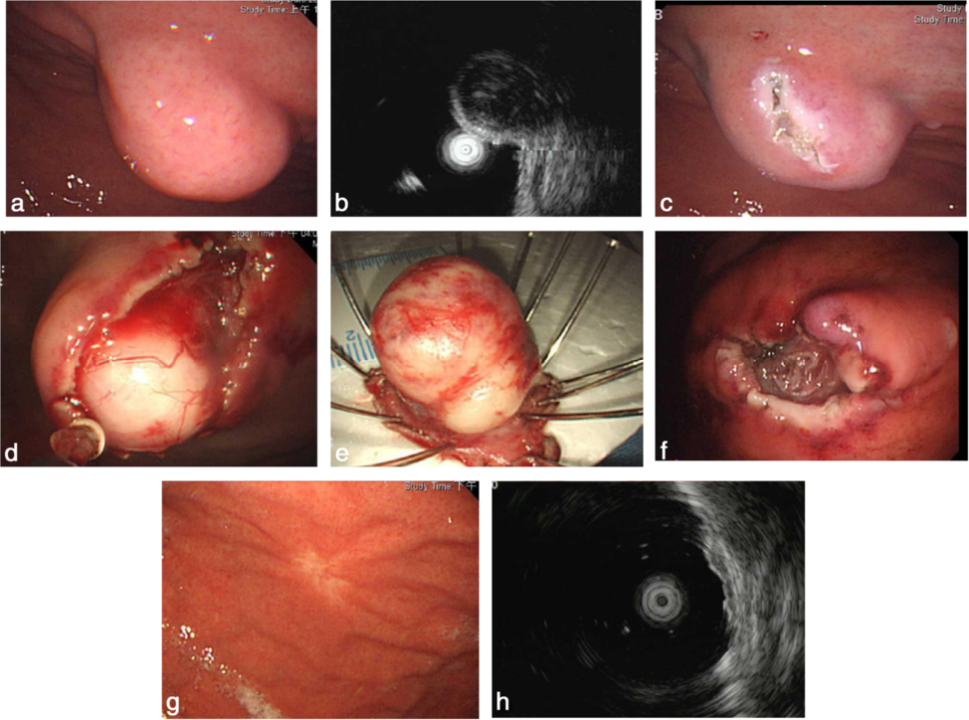
**A gastric subepithelial tumor originating from the muscularis propria layer was resected by the modified ESD with enucleation.a**. Endoscopic view of the gastric subepithelial tumor at gastric high body. **b**. Endoscopic ultrasonography (EUS) revealed an inhomogeneous hypoechoic tumor arising from the muscularis propria layer. **c**. A precut and longitudinal incision of the mucosa. **d**. Lateral dissection with an insulated-tip knife 2 exposing an encapsulated tumor. **e**. A slight dumbbell-shaped tumor was enucleated. **f**. No visible residual tumor at ulcer base. **g**. A whitish scar was shown 2 months after dissection. **h**. EUS revealed mild thickening of the second layer and no recurrent tumor in the muscularis propria layer.

**Figure 2:**
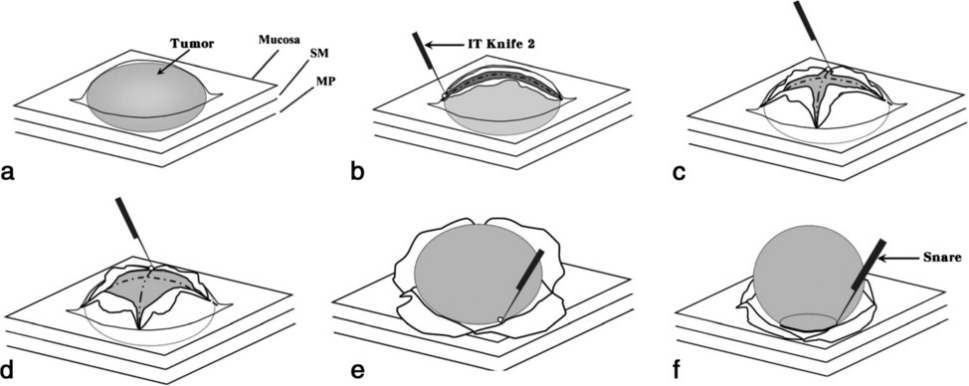
**Schematic illustration of the modified endoscopic submucosal dissection for subepithelial tumor originating from the muscularis propria layer. a**. Subepithelial tumor; SM (submucosa); MP (muscularis propria). **b**. A longitudinal incision made by insulated-tip knife 2. **c**. Transverse incision made (orange peeling method). **d**. Tumor exposed after lateral dissection. **e**. Tumor was finally dissected by insulated-tip knife 2, or **f**. By snare polypectomy (at the fundus).

All patients were hospitalized for ESD therapy under informed consent. Conscious sedation was provided by intravenous administration of midazolam and meperidine. The modified ESD technique was performed using a standard endoscope coupled with an auxiliary water jet (GIF-Q260J; Olympus Optical, Tokyo, Japan). The procedure (Figure 1) started by injecting 5 mL of normal saline into the submucosa layer at the proximal end of the subepithelial tumor to create a submucosal liquid pool. A precut was made with a needle knife (CD-1 L; Olympus Optical, Tokyo, Japan) at the injection site at 30w of PulseCut slow mode from an electrosurgical generator (ESG-100; Olympus Optical, Tokyo, Japan). The needle knife was then inserted to make a longitudinal incision first, followed by a transverse incision laterally to the sides of the tumor, like orange peeling (Figure 2). Thus, a well-demarcated encapsulated tumor was exposed. The insulated-tip knife 2 (IT-knife 2, KD-611 L; Olympus Optical, Tokyo, Japan) was placed to dissect the connective tissue between the tumor and the submucosa. When the submucosa was completely separated from the tumor, the underlying muscularis propria was dissected away to lift the tumor. Subsequently, the tumor was excised with the IT-knife 2. When the tumor was located at fundus, the final step of the dissection was performed with the technique of polypectomy (Figure 2f) by employing an electrocautery snare (SD-5U-1; Olympus Optical, Tokyo, Japan) energized at 40w. All tumors were retrieved by a net.

Complete resection was defined as absence of any tumor remnant on endoscopic view following resection (Figure 1f). Perforation was defined as a visible hole or extraluminal structure during the procedure or free air on plain abdominal film the next day. Overt bleeding was defined as bright red blood vomiting, tarry stool passage or a drop of hemoglobin level in 24 hours of ≥2 g/dl. Abdominal pain was evaluated with Visual Analogue Scale (VAS).

Tumor specimen was submitted for Hematoxylin and Eosin and immunohistochemical staining of CD 34, CD 117 (c-kit), smooth muscle actin (SMA), and S-100 marker. Mitotic index (number of mitosis under high-power field) was scored. Those tumors stained positive for both CD 34 and CD 117 and negative for SMA were diagnosed as GIST, those positive for SMA and negative for both CD 34 and CD 117 were diagnosed as leiomyoma, and those positive for S-100 were diagnosed as neurogenic tumor.

All patients were observed for 48 hours after ESD. A proton pump inhibitor was administered. All patients underwent endoscopic examination at 2 months later, EUS or CT scan examination every 6 months for one year, and every 12 months thereafter.

## Results

Patient's demographic characteristics, tumor location, endoscopic features, and treatment outcome are summarized in Table 1. The mean size of the subepithelial tumors as measured by EUS was 26.1 mm (range: 20–42 mm). Nine lesions were located at the gastric body, 3 at the fundus, 3 at the antrum and 1 at the cardia. The mean procedure time was 52 minutes (range: 30–120 minutes). Four tumors were pedunculated and 12 were sessile. Fifteen of the sixteen tumors were successful complete resection. Immunohistochemical study of these 15 tumors showed GIST in 13 patients and leiomyoma in 2 patients. Mitotic index was low (less than 5 mitosis per 50 HPFs) in all tumors. The only tumor that could not be completely resected was the largest one (42 mm). This tumor adhered tightly to the underlying muscularis propria layer. This patient was referred for surgical resection. The pathological diagnosis was a GIST with low mitotic index.

**Table 1:** Tumor characteristics and treatment outcomes of the modified endoscopic submucosal dissection with enucleation for gastric subepithelial tumors

**Case no.**	**Age(y)/Sex**	**Location**	**Endoscopic feature**	**Tumor Size (mm)**	**Complete resection**	**Procedure time (minutes)**	**Complication**	**Pathology/(mitosis/HPF**	**Follow-up period(mo)/Recurrence**
1	66/F	High body AW	Pedunculated	28	Y	45	N	GIST/<5/50	22/N
2	55/F	High body AW	Sessile	42	N	120	N	GIST/<5/50	21/N
3	49/F	High body AW	Sessile	24	Y	40	N	GIST/<5/50	21/N
4	35/M	Antrum AW	Sessile	22	Y	35	Pain	GIST/<5/50	20/N
5	47/M	High body AW	Pedunculated	20	Y	35	N	GIST/<5/50	19/N
6	53/F	Cardia	Sessile	25	Y	50	N	GIST/<5/50	18/N
7	65/M	Fundus	Sessile	30	Y	75	N	Leiomyoma	16/N
8	57/M	Middle body PW	Sessile	27	Y	45	N	GIST/<5/50	16/N
9	52/M	Middle body GC	Pedunculated	21	Y	30	Pain	GIST/<5/50	15/N
10	49/F	Antrum PW	Sessile	29	Y	50	N	GIST/<5/50	13/N
11	39/F	High body AW	Sessile	32	Y	55	N	Leiomyoma	12/N
12	51/F	Fundus	Sessile	24	Y	65	N	GIST/<5/50	11/N
13	56/F	Fundus	Sessile	20	Y	60	N	GIST/<5/50	10/N
14	59/M	High body AW	Sessile	25	Y	45	Pain	GIST/<5/50	9/N
15	47/F	High body AW	Sessile	21	Y	45	N	GIST/<5/50	8/N
16	55/F	Antrum PW	Pedunculated	27	Y	40	N	GIST/<5/50	6/N

GIST, gastrointestinal stromal tumor; AW, anterior wall; PW, posterior wall; GC, greater curvature.

Minor bleeding was observed during the procedure and was successfully managed in all cases with Coagrasper (FD-410LR; Olympus Optical, Tokyo, Japan) at 80w of SoftCoag mode. There was no procedure-related perforation. Three patients experienced mild epigastric pain (VAS of 2) after the procedure, and their symptom subsided within 2 days. During a mean follow-up duration of 14.8 months (range: 6–22 months), there was no residual or recurrent tumor found.

## Discussion and conclusion

Histological diagnosis of gastric subepithelial tumors is difficult. Endoscopic biopsy often fails to obtain adequate tissue from the deep layer of the stomach. EUS offers an alternative modality for diagnosis by showing its layer of origin, shape, border, size, and texture echogenicity. These parameters have been shown to predict its malignant potential of a GIST, with a sensitivity of 83%-86% and a specificity of 76%-80% [6,7]. In addition, EUS-guided fine needle aspiration for cytology (FNAC) [6] or Tru-cut biopsy [8] provides sufficient tissue for histopathological and immunohistochemical diagnosis. Polkowski et al. [9] reported a diagnostic yield of 67% (0%-94%) by EUS-FNAC, and 91% (87%-100%) by EUS-guided biopsy. However, calculation of mitotic index remains difficult using tissue core obtained from either needles, and sampling bias further confounds the interpretation.

**Figure 3:**
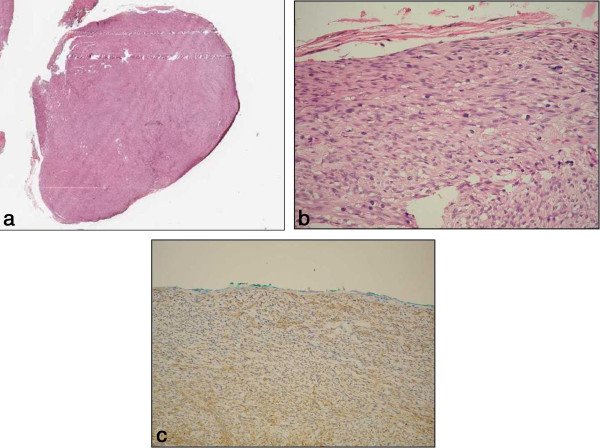
**Pathology of GIST after ESD with enucleation. a**. A well-defined tumor surrounded by a thin capsule (Hematoxylin-eosin, 1x). **b**. A spindle cell tumor surrounded by fibrous tissue (Hematoxylin-eosin, 400x). **c**. The tumor cells are positive for c-Kit with immunohistochemical stain (200x).

With recent advent of endoscopic resection techniques, endoscopists can now remove mucosal or submucosal tumors by endoscopic mucosal resection (EMR) [10,11]. Yet complete resection of subepithelial tumors larger than 2 cm in size and those originating from the muscularis propria layer remain difficult by EMR. Recently, ESD has been introduced for treatment of early cancers of gastrointestinal tract [5]. It allows complete resection of superficial lesions irregardless of their size. With the development of endoscopic resection techniques and devices, endoscopic treatment for tumors originating from the muscularis propria became possible. Park et al. [12] first demonstrated endoscopic enucleation of esophagogastric subepithelial tumors using IT-knife. Fourteen of the 15 (93.3%) subepithelial tumors were successfully resected. Of these tumors, eleven originated from the muscularis propria layer and 5 were GISTs. Shim et al. [11] described several tools for ESD with enucleation of subepithelial tumors originating from the muscularis propria layer, including electrosurgical snare, cutting knife and IT-knife. Lee et al. [13] achieved successful resection of 9 out of 12 gastric subepithelial tumors originating from the muscularis propria layer by IT-knife, while the remaining 3 were partially removed by EMR with a cap. GIST with low malignant potential was diagnosed in 8 cases and leiomyoma in 4 cases. In our study, fifteen of the 16 subepithelial tumors were resected completely in one piece by using a modified ESD with enucleation. The resection technique in this study was similar to that by Park et al. [12]. But instead of making a longitudinal incision alone, an additional transverse incision to the lateral sides of the tumor was made. This additional incision, like orange peeling, exposes the tumor and its underlying muscularis propria layer more clearly, so that complete dissection of the tumor can be more easily performed. The only tumor that could not be completely resected was the largest one of 42 mm in size, due to its wide contact area with the underlying muscularis propria layer, this patient received surgical treatment. The location of the tumor is a point of concern when performing this procedure. More time was consumed for resection of the tumor in the fundus than those in the body or the antrum. This is because retroflexion of the endoscope brings the IT knife 2 vertically oriented to the muscularis propria layer, dissection is more difficult as a result. In our case, we used the technique of polypectomy instead by employing an electrosurgical snare in the final step of tumor resection successfully. All the GISTs we removed were encapsulated which allowed complete dissection. Pathological examination at low-power field confirmed the presence of an intact and thin fibrous capsule (Figure 3).

The two common ESD-related complications are perforation and bleeding. Perforation risk following endoscopic resection of subepithelial tumors originating from the muscularis propria layer has been estimated from 0-28% [12-14]. The most common location of perforation is the fundus. Nevertheless, most of the perforations are small and can be successfully managed by endoscopic application of hemoclips and without need for surgical intervention. Most of the studies reported, no overt bleeding during endoscopic resection. In this study, there was a high success rate (15/16) for complete resection by the modified ESD with enucleation we introduced. No procedure-related perforation or overt bleeding occurred. Minor bleeding during the procedure was common but adequate hemostasis was always achieved. Epigastric pain was usually mild.

In Miettinen’s series, the size of GIST is between 2 and 5 cm, there is a 1.9% of tumor-related mortality or distant metastasis when mitotic index is less than or equal to 5 mitosis per 50 HPFs, and a 16% when mitotic index is higher than 5 mitosis per 50 HPFs [15]. Most of the gastric subepithelial tumors in our series were GISTs (14/16), their size ranges from 2 to 5 cm and they all had mitosis index of less than 5 per 50 HPFs. We followed our patients for a mean duration of 14.8 months. There was no local recurrence or distant metastasis. However, long-term follow-up is suggested.

In conclusion, we introduce a modified ESD with enucleation for complete resection of gastric subepithelial tumors originating from the muscularis propria layer and larger than 2 cm. This procedure preserves the integrity of the stomach and shortens hospital stay. It is a safe and effective alternative to surgical therapy.

## Abbreviations

ESD: Endoscopic Submucosal Dissection; GIST: Gastrointestinal Stromal Tumors; EUS: Endoscopic Ultrasonography; EUS-FNAC: EUS-guided Fine Needle Aspiration for Cytology; EMR: Endoscopic Mucosal Resection; CT: Computed tomography; IT-knife 2: Insulated-tip knife 2.

## Competing interests

The authors declare no conflicting interests.

## Author’s contributions

C-YY, L-JM participated in writing the manuscript. T-MH and N-SC participated in study design. C-CT participated in data analysis and interpretation. C-TC participated in pathological interpretation. Y-KC helped with drafting the manuscript. C-YY, L-JM and N-SC approved the final manuscript.

## Pre-publication history

The pre-publication history for this paper can be accessed here:

http://www.biomedcentral.com/1471-230X/12/124/prepub
